# Pre-pregnancy and pregnancy disorders, pre-term birth and the risk of cerebral palsy: a population-based study

**DOI:** 10.1093/ije/dyad106

**Published:** 2023-07-26

**Authors:** Neda Razaz, Sven Cnattingius, Sarka Lisonkova, Shahrzad Nematollahi, Maryam Oskoui, K S Joseph, Michael Kramer

**Affiliations:** Clinical Epidemiology Division, Department of Medicine Solna, Karolinska Institutet, Stockholm, Sweden; Clinical Epidemiology Division, Department of Medicine Solna, Karolinska Institutet, Stockholm, Sweden; Department of Obstetrics and Gynaecology, University of British Columbia and the Children’s and Women’s Hospital and Health Centre of British Columbia, Vancouver, BC, Canada; School of Physical and Occupational Therapy, McGill University, Montreal, QC, Canada; Department of Clinical Research, Shriners Hospitals for Children, Montreal, QC, Canada; Child Health and Human Development Program, Centre for Outcomes Research and Evaluation, McGill University Health Center Research Institute, Montreal, QC, Canada; Department of Pediatrics, Faculty of Medicine and Health Sciences, McGill University, Montreal, QC, Canada; Department of Neurology & Neurosurgery, Faculty of Medicine and Health Sciences, McGill University, Montreal, QC, Canada; Department of Obstetrics and Gynaecology, University of British Columbia and the Children’s and Women’s Hospital and Health Centre of British Columbia, Vancouver, BC, Canada; School of Population and Public Health, University of British Columbia, Vancouver, BC, Canada; Department of Pediatrics, Faculty of Medicine, McGill University, Montreal, QC, Canada; Department of Epidemiology, Biostatistics and Occupational Health, Faculty of Medicine, McGill University, Montreal, QC, Canada

**Keywords:** Cerebral palsy, maternal chronic illness, pre-term birth, mediation analysis

## Abstract

**Background:**

Cerebral palsy (CP) is the most common cause of childhood physical disability whose aetiology remains unclear in most cases. Maternal pre-existing and pregnancy complications are recognized risk factors of CP but the extent to which their effects are mediated by pre-term birth is unknown.

**Methods:**

Population-based cohort study in Sweden including 2 055 378 singleton infants without congenital abnormalities, born between 1999 and 2019. Data on maternal and pregnancy characteristics and diagnoses of CP were obtained by individual record linkages of nationwide Swedish registries. Exposure was defined as maternal pre-pregnancy and pregnancy disorders. Inpatient and outpatient diagnoses were obtained for CP after 27 days of age. Adjusted rate ratios (aRRs) were calculated, along with 95% CIs.

**Results:**

A total of 515 771 (25%) offspring were exposed to maternal pre-existing chronic disorders and 3472 children with CP were identified for a cumulative incidence of 1.7 per 1000 live births. After adjusting for potential confounders, maternal chronic cardiovascular or metabolic disorders, other chronic diseases, mental health disorders and early-pregnancy obesity were associated with 1.89-, 1.24-, 1.26- and 1.35-times higher risk (aRRs) of CP, respectively. Most notably, offspring exposed to maternal antepartum haemorrhage had a 6-fold elevated risk of CP (aRR 5.78, 95% CI, 5.00–6.68). Mediation analysis revealed that ∼50% of the effect of these associations was mediated by pre-term delivery; however, increased risks were also observed among term infants.

**Conclusions:**

Exposure to pre-existing maternal chronic disorders and pregnancy-related complications increases the risk of CP in offspring. Although most infants with CP were born at term, pre-term delivery explained 50% of the overall effect of pre-pregnancy and pregnancy disorders on CP.

Key Messages

- Pre-existing maternal chronic conditions and pregnancy-related complications were associated with an increased risk of cerebral palsy (CP) in children, particularly among those who were exposed to maternal chronic cardiovascular or metabolic disorders, pre-eclampsia or antepartum haemorrhage.
- Approximately 50% of the effects of these disorders was mediated by pre-term delivery; however, increased risks were also observed among term infants.
- The significant increase in CP risk associated with chronic conditions and pregnancy disorders suggests biological pathways and mechanisms that may lead to CP.

## Introduction

Cerebral palsy (CP) refers to a motor disability of cerebral origin accompanied by a lifelong movement disorder affecting between 1.5 and 3 per 1000 live births in the world.^1^^,^^2^ CP carries a significant lifetime burden for affected children and their families, owing to motor and intellectual disability, epilepsy and mental health disorders^3^ and a shortened life expectancy, particularly in individuals with severe motor and eating impairments.^4^

Pre-term birth is strongly associated with CP, with a marked increase in risk with decreasing gestational age at birth.^5^^,^^6^ Neonatal morbidity, such as birth asphyxia, has been implicated in the aetiology of cerebral palsy,^7^ especially in infants born pre-term, whereas other neurological neonatal diseases play the leading role from 34 weeks onwards.^6^ Paradoxically, however, it has long been recognized that the majority of children with CP are born at term.^8^ This knowledge led to the former belief that CP could be prevented by improvements in obstetric and neonatal care, but that belief has now been tempered by more recent studies documenting the high risks of CP associated with congenital malformations, fetal growth restriction and maternal obesity.^8^^,^^9^ A recent population-based Norwegian study reported that several chronic maternal disorders, including both type 1 and type 2 diabetes and autoimmune disorders such as lupus and Crohn’s disease, are significantly associated with CP in offspring.^10^ Other maternal chronic diseases, mental illness and pregnancy complications like gestational diabetes, gestational hypertension and pre-eclampsia and antepartum haemorrhage have been less well explored.^7–9^

Placental dysfunction associated with hypertensive disorders may result in reduced placental perfusion and oxidative stress,^11^ leading to inadequate fetal growth, suboptimal nutrient and oxygen availability for the fetus, brain injury or developmental impairment.^12^^,^^13^ Many pre-existing and pregnancy complications are also associated with pre-term birth, a strong risk factor for CP, although the pathophysiological mechanisms remain unknown. Thus, pre-term birth lies on the causal pathway between maternal disorders and CP, and adjustment for gestational age introduces an ‘overadjustment’ bias,^14^^,^^15^ which is a major limitation of previous studies.^9^^,^^10^^,^^16–18^

In this population-based study, we used data on >2 million pregnancies to examine the associations between pre-existing maternal chronic disorders and pregnancy-related complications and the risk of CP in offspring. We also assessed the extent to which pre-term delivery and restricted fetal growth mediate the effects of these maternal disorders on CP. Moreover, because CP can be diagnosed only if the fetus is born alive and survives the neonatal period, we analysed stillbirth and neonatal death as competing risks for CP.

## Methods

Our study was based on singleton births in Sweden between 1999 and 2019 recorded in the Medical Birth Register.^19^ This database contains information on antenatal, obstetric and neonatal care that is prospectively recorded on standardized forms on >98% of all births in Sweden. Using the person-unique national registration numbers of mothers and infants, data from the Birth Register can be linked to several national registries. The nationwide National Patient Register^20^^,^^21^ includes diagnostic codes and procedures on hospital inpatient care since 1987 and hospital outpatient care from 2001. The Cause of Death Register includes information on all deaths in Sweden.^22^ In the Medical Birth-, Patient-, and Cause of Death Registers, maternal diseases diagnosed prior to delivery and neonatal death are coded using the Swedish version of the International Classification of Diseases, the ninth revision (ICD-9) from 1987 through 1996, and the tenth revision (ICD-10) since 1997. Information on maternal education and country of origin was obtained from the Education Register and the Total Population Register, respectively.^23^^,^^24^

### Study population

Between 1 January 1999 and 31 December 2019, we identified 2 146 210 singleton live births and stillbirths at ≥22 completed gestational weeks from the Medical Birth Register with valid personal identification numbers. We excluded infants with major malformation (*n* = 87 827), those with missing information on gestational age or maternal age (*n* = 1058) and those born at ≥44 + 0 weeks (*n* = 1947). The final study population included 2 055 378 singleton infants without congenital abnormalities.

### Pre-pregnancy and pregnancy disorders

Relevant maternal pre-existing (chronic) disorders were identified based on a review of the literature^25^ and women with such conditions diagnosed at any time prior to conception (calculated as the date of delivery minus gestational age) were identified using ICD-9 and ICD-10 codes (Supplementary Table S1, available as Supplementary data at *IJE* online).^25^ We categorized maternal pre-existing chronic disorders as cardiovascular and/or metabolic disorders (i.e. chronic heart disease, cerebrovascular diseases, ischaemic heart disease, insulin-dependent and non-insulin-dependent diabetes mellitus and chronic hypertension), mental disorders (i.e. anxiety and stress-related disorders, depression and other mood disorders, eating disorder, bipolar disorder, schizophrenia and other psychotic disorders) and other chronic disorders (i.e. asthma, thyroid disorder, inflammatory bowel disease, epilepsy, multiple sclerosis, migraine, celiac disease, rheumatoid arthritis, lupus erythematosus and chronic kidney disease). As shown in Supplementary Tables S1 and Table S2 (available as Supplementary data at *IJE* online), pregnancy-related complications included, early-pregnancy obesity [i.e. body mass index (BMI) of ≥30 kg/m^2^], gestational diabetes, pre-eclampsia and antepartum haemorrhage (the latter including placenta praevia, placental abruption and other antepartum bleeding). Maternal BMI in early pregnancy was calculated from self-reported height and weight measured in light clothing at the first prenatal visit, which occurred within the first 14 weeks of gestation for 90% of pregnant women.^19^ Pre-eclampsia included eclampsia and early- and late-onset pre-eclampsia.

### Outcome

CP was identified by the presence of diagnostic codes for CP (ICD-10 code G80), either in hospitalization or outpatient visit records after 27 days of age. The quality of CP diagnoses in Sweden is high, with the majority of included cases from country regions with well-established population-based CP registries. Paediatric neurologists who are experts on CP systematically validate the cases through medical record review and/or physical examination.^9^ The date associated with the first record of CP was considered the date of diagnosis. A composite outcome of death or CP (i.e. stillbirth, infant death or CP) was also examined.

### Covariates

Gestational age (in completed weeks) was estimated using the following hierarchy: the date of early second-trimester ultrasound (87.7%), the date of the last menstrual period (7.4%) and a post-natal assessment (4.9%). We defined pre-term delivery as gestational age between 22 and 36 completed weeks. We calculated z-scores of birthweight-for-gestational age based on the ultrasound-based, sex-specific Swedish reference curve for fetal growth.^26^ The z-scores were further converted into birthweight-for-gestational age percentiles. Small-for-gestational age was defined as birthweight-for-gestational age of <10th percentile. Maternal characteristics examined included age at delivery, country of birth, education, cohabitation with a partner, parity, height, BMI and smoking during pregnancy. Mothers who reported daily smoking at the first antenatal visit and/or at 30–32 gestational weeks were classified as smokers.

### Statistical analysis

**Figure 1:**
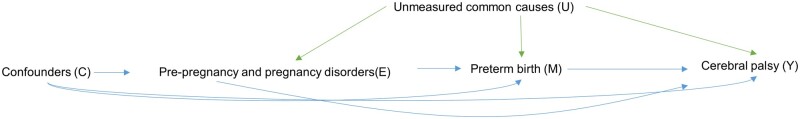
Simplified directed acyclic graph depicting the relationship between pre-existing and pregnancy disorders and cerebral palsy with pre-term birth as the mediator and unmeasured confounders

We assessed the distribution of maternal characteristics by overall maternal pre-existing chronic disorders status (i.e. pre-existing cardiovascular, metabolic or mental disorders or other chronic disorders). To assess the associations between maternal disorders and CP, we estimated risk ratios (RRs) with 95% CIs, derived from fitting log-linear Poisson regression models with robust variance. Confounders were included in the final model based on the literature^6^ and our directed acyclic graph (Figure 1). In the multivariable analyses, estimates were adjusted for maternal age, parity, mother’s country of origin, education level, cohabitation with partner, smoking, child’s sex and year of delivery.

To account for the correlation among siblings, we used a robust sandwich estimator of variance in all models. Furthermore, since death prior to the diagnosis of CP is a competing risk for CP, we additionally quantified the adjusted association between maternal disorders and a composite outcome of death or CP (i.e. stillbirth, infant death or CP). Log-linear Poisson regression models were used to assess the association between maternal disorders and the composite outcome, adjusting for the same confounders as in the main analysis. The population-attributable fractions (PAFs) for CP associated with a maternal disorders (or, in other words, the fraction of CP that could be prevented by eliminating a specific maternal disorder) were estimated using the following formula^2^,^7^:


PAF=p∗(RR−1)/RR,

where PAF denotes the population-attributable fraction, *p* denotes the proportion of the population exposed to a specific maternal disorder and RR denotes the rate ratio contrasting the rate of CP among infants with a specific maternal disorder vs those without that specific maternal disorder.

### Causal mediation analysis

Gestational age and small-for-gestational age (SGA) at birth, a proxy for intrauterine growth restriction, may lie on the causal pathway between maternal disorders and CP (see Figure 1 and Supplementary Table S3, available as Supplementary data at *IJE* online) and thus any adjustment for gestational age or SGA will bias the resulting associations with those antecedent risk factors. We therefore undertook causal mediation analyses based on a counterfactual framework^28^ using the generalized linear Poisson regression models to disentangle the associations between maternal disorders and CP into the natural direct effect (the effect between maternal disorders and CP in the absence of pre-term delivery or SGA) and the natural indirect effect (the effect operating through pre-term delivery or SGA). We also estimated the controlled direct effect, which estimates the effect of maternal disorders on CP that is not mediated through pre-term delivery (i.e. among term births) or SGA. We additionally assessed the proportions of the total effect between maternal disorders and CP that was mediated through pre-term delivery and SGA. Factors that were not found to be mediators in the initial analysis were excluded from the final mediation analyses. Log-linear regression models and the mediation analysis were carried out in SAS (version 9.4; SAS Institute, Cary, NC, USA) using GENMOD and the CAUSALMED procedures, respectively.

### Sensitivity analyses

We performed several sensitivity analyses. First, given that mediation methods were developed under a strict assumption of no unmeasured confounding,^28^ we examined the robustness of causal effects to unmeasured confounders by estimating an E-value. The E-value provides an estimate of the smallest excess risk of the unmeasured confounder(s) beyond that accounted for the adjusted confounders to draw the observed excess towards the null. In addition, the E-value provides an indication of the smallest excess risk of the unmeasured confounder to move the lower 95% CI of the observed excess risk to cross the null.^29^ Second, since some of the covariates had missing values, we imputed missing data through multiple imputation using a chained equations approach.^30^ We assumed that the pattern of missing data was ‘missing at random’ and created 10 imputed data sets (after 200 burn-in iterations). Fourth, to account for the varying lengths of follow-up (the probability of being diagnosed with CP increases with age), we calculated hazard ratios (HRs) and 95% CIs using of Cox proportional hazard models with a time axis starting at 1 year of age. Fifth, to ensure that the associations were robust to maternal pre-existing conditions diagnosed closer to the index pregnancy, we repeated the main analysis restricting the pre-existing chronic disorders to those diagnosed within 5 years before conception.

## Results

Of 2 055 378 live-born and stillborn fetuses delivered between 1999 and 2019, 515 771 (25%) were born to mothers with pre-existing chronic disorders. Mothers with pre-existing disorders were more likely to be older (>40 years), grand multiparous (parity of ≥4), to smoke, to live alone, to be born in Sweden and to have a lower educational level (Table 1). The rates of pre-term delivery (<37 weeks) were 5.9% and 4.3% in women with and without any pre-existing chronic disorders, respectively. Anxiety disorders, depression, asthma and thyroid disorders were among the most common pre-existing conditions during pregnancy (Supplementary Table S2, available as Supplementary data at *IJE* online).

**Table 1:** Distribution of maternal characteristics and maternal pre-existing chronic disorders, singleton, non-malformed births 1999–2019, Sweden

Maternal characteristics	Number of deliveries	Maternal pre-existing chronic disorders	
		No	Yes
	*n* = 2 055 378	*n* = 1 539 607	*n* = 515 771
	*n* (%)	*n* (%)	*n* (%)
Maternal age (years)			
≤19	29 178 (1.4)	22 110 (1.4)	7068 (1.4)
20–29	889 283 (43.3)	665 741 (43.2)	223 542 (43.3)
30–34	710 095 (34.5)	543 882 (35.3)	166 213 (32.2)
35–39	351 972 (17.1)	257 422 (16.7)	94 550 (18.3)
40–44	71 203 (3.5)	48 191 (3.1)	23 012 (4.5)
≥45	3647 (0.2)	2261 (0.1)	1386 (0.3)
Parity			
1	901 574 (43.9)	697 766 (45.3)	203 808 (39.5)
2	761 251 (37.0)	570 663 (37.1)	190 588 (37.0)
3	274 436 (13.4)	197 781 (12.8)	76 655 (14.9)
4	118 117 (5.7)	73 397 (4.8)	44 720 (8.7)
Maternal height (cm)			
Missing	36 004 (1.8)	30 781 (2.0)	5223 (1.0)
≤159	277 458 (13.5)	203 875 (13.2)	73 583 (14.3)
160–164	515 426 (25.1)	381 279 (24.8)	134 147 (26.0)
165–169	585 794 (28.5)	438 762 (28.5)	147 032 (28.5)
≥170	640 696 (31.2)	484 910 (31.5)	155 786 (30.2)
Maternal education			
Missing	19 560 (1.0)	15 292 (1.0)	4268 (0.8)
≤9	173 728 (8.5)	113 255 (7.4)	60 473 (11.7)
10–11	228 548 (11.1)	163 725 (10.6)	64 823 (12.6)
12	529 390 (25.8)	376 789 (24.5)	152 601 (29.6)
13–14	294 387 (14.3)	224 054 (14.6)	70 333 (13.6)
≥15	809 765 (39.4)	646 492 (42.0)	163 273 (31.7)
Mother cohabits with partner			
Missing	100 358 (4.9)	80 993 (5.3)	19 365 (3.8)
Yes	183 6126 (89.3)	1 380 273 (89.7)	455 853 (88.4)
No	118 894 (5.8)	78 341 (5.1)	40 553 (7.9)
Country of birth			
Missing	1979 (0.1)	1401 (0.1)	578 (0.1)
Sweden	1 575 228 (76.6)	1 161 644 (75.5)	413 584 (80.2)
Other Nordic	31 633 (1.5)	24 946 (1.6)	6687 (1.3)
Non-Nordic	446 538 (21.7)	351 616 (22.8)	94 922 (18.4)
Smoking during pregnancy			
Missing	77 854 (3.8)	65 390 (4.2)	12 464 (2.4)
No	1 825 055 (88.8)	1 375 981 (89.4)	449 074 (87.1)
Yes	152 469 (7.4)	98 236 (6.4)	54 233 (10.5)
Year of delivery			
1999–2003	425 797 (20.7)	365 387 (23.7)	60 410 (11.7)
2004–07	378 964 (18.4)	304 919 (19.8)	74 045 (14.4)
2008–11	407 944 (19.8)	305 017 (19.8)	102 927 (20.0)
2012–15	416 968 (20.3)	289 238 (18.8)	127 730 (24.8)
2016–19	425 705 (20.7)	275 046 (17.9)	150 659 (29.2)
Pre-term delivery (<37 weeks)			
No	1 959 297 (95.3)	1 474 057 (95.7)	485 240 (94.1)
Yes	96 081 (4.7)	65 550 (4.3)	30 531 (5.9)
Small-for-gestational age at birth			
No	1 925 590 (93.7)	1 441 891 (93.7)	483 699 (93.8)
Yes	129 788 (6.3)	97 716 (6.3)	32 072 (6.2)

We observed 3472 children with CP for a cumulative incidence of 1.7 per 1000 total births (Table 2). The median age at diagnosis was 2.0 years (interquartile range: 1.1–3.7 years). Among these infants, 766 (20%) children were diagnosed before 1 year of age and 1011 (30%) before 2 years. The incidence of CP was highest among infants exposed to maternal chronic cardiovascular or metabolic disorders, mental disorders, obesity, pre-eclampsia and antepartum haemorrhage. After adjusting for potential confounders, an increase in the risk of CP was seen in all pregnancies with pre-existing and pregnancy disorders, except for those with gestational diabetes (Table 2). Maternal chronic cardiovascular or metabolic disorders, other chronic disorders, mental disorders and obesity were associated with 89%, 24%, 26% and 35% increases in the risk of CP, respectively. Most notably, offspring exposed to maternal antepartum haemorrhage had a 6-times higher risk of CP compared with those not exposed to maternal antepartum haemorrhage. Overall, the PAF was 3.7% for obesity, 3.2% for pre-eclampsia, 5.3% for antepartum haemorrhage and <2% for other chronic disorders, mental disorders and cardiovascular and metabolic disorders (Table 2). Analyses of associations between maternal disorders and composite death or CP showed similar effects except that the association between mental disorders and composite death or CP was not statistically significant (Table 3).

**Table 2:** Pre-existing maternal chronic disorders and pregnancy-related complications and rates of cerebral palsy among live-born singleton births without congenital abnormalities in Sweden, 1999–2019

Maternal disorders	No. of children	No. with CP	Rate per 1000 births	Relative risk (95% CI)		
				Model 1^e^	Model 2^f^	PAF
Overall	2 055 378	3472	1.69			
Chronic cardiovascular and metabolic disorders^a^	38 470	128	3.33	2.00 (1.68–2.39)	1.89 (1.55–2.29)	0.9
Other chronic disorders^b^	177 105	320	1.81	1.08 (0.96–1.21)	1.24 (1.10–1.41)	1.7
Mental disorders^c^	159 341	305	1.91	1.14 (1.02–1.29)	1.26 (1.10–1.43)	1.6
Obesity (BMI ≥30 kg/m^2^)	234 588	499	2.13	1.30 (1.18–1.43)	1.35 (1.22–1.49)	3.0
Gestational diabetes	26 695	55	2.06	1.22 (0.93–1.59)	1.23 (0.92–1.63)	0.2
Pre-eclampsia	55 670	210	3.77	2.31 (2.01–2.65)	2.14 (1.84–2.48)	1.4
Antepartum haemorrhage^d^	22 375	221	9.88	6.14 (5.36–7.04)	5.78 (5.00–6.68)	0.9

CP, cerebral palsy; BMI, body mass index.

a Chronic cardiovascular and metabolic disorders: any of type 1 and 2 diabetes, ischaemic heart disease, chronic heart disease, cerebrovascular diseases and chronic hypertension.

b Other chronic disorders include: asthma, thyroid disorder, inflammatory bowel disease, epilepsy, multiple sclerosis, migraine, celiac disease, rheumatoid arthritis, lupus erythematosus and chronic kidney disease.

c Mental disorders includes any of anxiety and stress-related disorders, depression and other mood disorders, eating disorders, bipolar disorders, schizophrenia and other psychotic disorders.

d Antepartum haemorrhage includes: placenta praevia, placental abruption and other antepartum bleeding.

e Unadjusted model.

f Model 2 adjusted for maternal age at childbirth, parity, educational level, country of birth, smoking during pregnancy, cohabitation with a partner, year of delivery and child's sex.

PAF denotes the population-attributable fraction and expresses the fraction of CP that could be prevented by eliminating a specific maternal disorder. Relative risk extracted from log-linear Poisson regression.

**Table 3:** Pre-existing maternal chronic disorders and pregnancy-related complications and rates of composite outcome among live-born singleton births without congenital abnormalities in Sweden, 1999–2019

		Composite outcome (stillbirth or neonatal death or cerebral palsy)			
Maternal disorders	No. of children	No. with CP	Rate per 1000 births	Relative risk (95% CI)	
				**Model 1** ^e^	**Model 2** ^f^
Overall	2 055 378	13 353	6.50		
Chronic cardiovascular and metabolic disorders^a^	38 470	551	14.32	2.23 (2.04–2.43)	2.12 (1.93–2.33)
Mental disorders^b^	159 341	1130	7.09	1.06 (0.99–1.13)	1.02 (0.96–1.10)
Other chronic disorders^c^	177 105	1236	6.98	1.08 (1.02–1.14)	1.15 (1.08–1.23)
Obesity (BMI ≥30 kg/m^2^)	234 588	2328	9.92	1.63 (1.55–1.70)	1.67 (1.59–1.75)
Gestational diabetes	26 695	194	7.27	1.09 (0.94–1.26)	0.98 (0.84–1.15)
Pre-eclampsia	55 670	673	12.09	1.90 (1.76–2.06)	1.71 (1.57–1.87)
Antepartum haemorrhage^d^	22 375	1215	54.30	9.09 (8.57–9.63)	8.27 (7.75–8.81)

CP, cerebral palsy; BMI, body mass index.

a Chronic cardiovascular and metabolic diseases: any of type 1 and 2 diabetes, ischaemic heart disease, chronic heart disease, cerebrovascular diseases and chronic hypertension.

b Mental disorders includes any of anxiety and stress-related disorders, depression and other mood disorders, eating disorders, bipolar disorders, schizophrenia and other psychotic disorders.

c Other chronic disorders include: asthma, thyroid disorder, inflammatory bowel disease, epilepsy, multiple sclerosis, migraine, celiac disease, rheumatoid arthritis, lupus erythematosus and chronic kidney disease.

d Antepartum haemorrhage includes: placenta praevia, placental abruption and other antepartum bleeding.

e Unadjusted model.

f Model 2 adjusted for maternal age at childbirth, parity, educational level, country of birth, smoking during pregnancy, cohabitation with a partner, year of delivery and child's sex.

SGA status at birth was not a mediator in the initial investigation (except mediating 42% of the total effect of pre-eclampsia on CP) and was excluded from the final mediation analysis. Table 4 shows the impact of pre-term delivery (<37 weeks) on the effect of maternal pre-existing and pregnancy disorders on CP. The RRs for the natural direct and natural indirect (mediated) effects of maternal chronic cardiovascular or metabolic disorders on CP were 1.46 (95% CI 1.14–1.78) and 1.29 (95% CI 1.17–1.41), respectively (Table 4). These findings indicate that 48% of the total effect of maternal chronic cardiovascular or metabolic disorders on CP was mediated by pre-term delivery. The proportion of excess CP following other chronic disorders, mental disorders, obesity, pre-eclampsia and antepartum haemorrhage that were mediated through pre-term delivery were estimated to be 36%, 33%, 14%, 55% and 59%, respectively (Table 4). The RRs for the controlled direct effects showed that all maternal disorders significantly increased the risk of CP in term infants, although other chronic and mental disorders were borderline statistically significant.

**Table 4:** Estimates of direct and indirect effects mediated through pre-term delivery (<37 weeks) on the association between pre-existing maternal chronic disorders and pregnancy-related complications and cerebral palsy live-born singleton infants in Sweden, 1999–2019

	Risk ratio (95% CI)				Percentagemediated
	Maternal disorders	Total effect	Controlled direct effect	Natural direct effect	Natural indirect effect
Chronic cardiovascular and metabolic disorders^a^	1.88 (1.52–2.25)	1.58 (1.17–1.98)	1.46 (1.14–1.78)	1.29 (1.17–1.41)	48
Other chronic disorders^b^	1.24 (1.08–1.39)	1.15 (0.98–1.31)	1.15 (1.01–1.30)	1.07 (1.06–1.09)	36
Mental disorders^c^	1.25 (1.09–1.41)	1.16 (0.98–1.34)	1.17 (1.02–1.32)	1.07 (1.05–1.09)	33
Obesity (BMI ≥30 kg/m^2^)	1.36 (1.22–1.49)	1.40 (1.24–1.56)	1.31 (1.18–1.44)	1.04 (1.03–1.05)	14
Pre-eclampsia	2.13 (1.82–2.45)	1.68 (1.33–2.03)	1.51 (1.24–1.79)	1.41 (1.27–1.55)	55
Antepartal haemorrhage^d^	5.77 (4.93–6.61)	3.17 (2.30–4.03)	2.94 (2.26–3.63)	1.96 (1.62–2.30)	59

BMI, body mass index.

Risk ratios are adjusted for maternal age at childbirth, parity, educational level, country of birth, smoking during pregnancy, cohabitation with a partner, year of delivery and child's sex.

a Chronic cardiovascular and metabolic diseases: any of type 1 and 2 diabetes, ischaemic heart disease, chronic heart disease, cerebrovascular diseases and chronic hypertension.

b Other chronic disorders include: asthma, thyroid disorder, inflammatory bowel disease, epilepsy, multiple sclerosis, migraine, celiac disease, rheumatoid arthritis, lupus erythematosus and chronic kidney disease.

c Mental disorders includes any of anxiety and stress-related disorders, depression and other mood disorders, eating disorders, bipolar disorders, schizophrenia and other psychotic disorders.

d Antepartum hemorrhage includes: placenta previa, placental abruption, and other antepartum bleeding.

### Sensitivity analyses

We performed several sensitivity analyses. First, the E-values for the RR and the lower limit of the 95% CI were greater in magnitude than the observed estimates for all maternal conditions (Supplementary Table S4, available as Supplementary data at *IJE* online), suggesting that the casual mediation parameters are robust to unmeasured confounding. HRs from the Cox regression analyses were similar to the RRs, indicating that the effect of follow-up time was minimal (Supplementary Table S5, available as Supplementary data at *IJE* online). Multiple imputation of missing data (Supplementary Table S6, available as Supplementary data at *IJE* online) yielded results consistent with those obtained in the main analysis. Finally, restricting maternal chronic disorders to those diagnosed within 5 years prior to pregnancy did not change the estimates (Supplementary Table S7, available as Supplementary data at *IJE* online). Inclusion of infants with congenital malformation did not change the estimates (data not shown).

## Discussion

In this nationwide cohort study, we found that exposure to pre-existing maternal chronic disorders and pregnancy-related complications increases the risk of CP in offspring. In particular, an elevated risk of CP was observed for chronic cardiovascular or metabolic disorders, mental disorders, obesity, pre-eclampsia and antepartum haemorrhage, although the PAF associated with these factors was small. Causal mediation analysis indicates that pre-term delivery explained 14% (obesity) to 59% (antepartal haemorrhage) of the effect of maternal disorders on CP. The controlled direct effects, however, suggest that chronic cardiovascular or metabolic disorders, obesity, pre-eclampsia and antepartum haemorrhage also directly increase the risk of CP among term infants.

Previous studies have shown an association between maternal chronic conditions, in particular autoimmune conditions, obesity, diabetes, pre-eclampsia, placenta praevia and CP.^9^^,^^10^^,^^16–18^ However, to our knowledge, ours is the first study to examine the role of pre-term delivery as a mediator of the effects of these maternal disorders on CP. Since chronic and pregnancy disorders may also cause pre-term birth, pre-term delivery is a mediator on the causal pathways between antecedent maternal disorders or pregnancy complications and CP.

It is important to stress that pre-term birth does not confound the association between maternal disorders and CP. In other words, adjustment or stratification based on pre-term delivery status introduces an ‘overadjustment’ bias^14^^,^^15^ that substantially underestimates the magnitude of the effects of maternal disorders. Previous studies have either adjusted for or stratified by pre-term birth,^9^^,^^10^^,^^16–18^ thus systematically underestimating the magnitude of associations between maternal disorders and CP. In our study, elevated risk of CP was also observed among term infants exposed to maternal chronic conditions and pregnancy disorders, which is in line with previous studies reporting increased risks of CP among term infants exposed to pre-eclampsia^31^ and chorioamnionitis.^32^ Furthermore, SGA status at birth was only an important mediator for the association between pre-eclampsia and CP.

Our results showed a marked increase in the prevalence of pre-existing chronic disorders during pregnancy over the last 20 years. This is consistent with a study demonstrating rising prevalence of chronic hypertension and diabetes during childbirth between 1989 and 2018 in the USA.^33^ The greatest increase in the risk of CP that we observed was in offspring of mothers with antepartum haemorrhage. In our study, cases of placental abruption constituted the majority of women with antepartum haemorrhage (125/221, 57%). Unfortunately, we were not able to determine the timing of antepartum haemorrhage in relation to delivery. Given the strong controlled and natural direct effects (3-fold increases in risk observed between antepartum haemorrhage and CP in term infants; see Table 4), it is plausible that placenta praevia and/or placental abruption may pose serious risks to fetal brain development.^8^^,^^34^ Most pre-term births are consequences of underlying pathological conditions and increasing evidence suggests that CP is often associated with longstanding intrauterine pathology such as bacterial or viral intrauterine infection or intrauterine growth restriction, which may also contribute to delivery complications and pre-term birth.^8^^,^^35^ Some prenatal factors may initiate a causal chain of events leading to CP through later pregnancy complications, e.g. maternal obesity via gestational hypertension or pre-eclampsia via placental abruption.

Overall, the population fraction of CP attributable to maternal disorders is small, with antepartum haemorrhage contributing to 5% of CP and other chronic and pregnancy disorders contributing to 1–3% of CP among singleton live births without congenital abnormalities. In other words, among 1000 singleton live births with CP without congenital abnormalities, only ∼50 occurrences of CP would be prevented if all pre-pregnancy and pregnancy disorders were eliminated. Although this estimate of the PAF is surprisingly small, the significant increase in CP risk associated with chronic conditions and pregnancy disorders may suggest biological pathways and mechanisms that lead to CP and also preventive interventions.^10^

Our study has several strengths. The population-based study design, together with the prospectively and independently collected information on maternal conditions and CP and other high-quality registry data,^21^ helps to minimize measurement bias and selection bias due to systematic losses to follow-up. Using data on >2 million pregnancies, we were able to identify associations between relatively rare exposures and the relative infrequent outcome. In addition, given that CP could be missed if those at high risk of CP die before diagnosis, we quantified the effects on a composite outcome that included stillbirth, neonatal death and CP, thereby addressing the issues related to outcome-dependent censoring and competing risks.^36^ Lastly, our hazard ratio estimates suggest that prolonged follow-up time does not affect time to CP diagnosis; most infants with CP, regardless of gestational age, are diagnosed early in infancy.

Nonetheless, our study also has some limitations. We lacked information on disease severity, duration and treatment, all of which could be informative in understanding the underlying causal mechanisms by which maternal disorders affect offspring risk of CP. Our ascertainment of maternal conditions may have been limited to more severe cases and resulted in an underestimation of their prevalence. Unmeasured confounding may bias our effect estimates, although the E-value estimates based on our sensitivity analyses suggest that the degree of this bias would have to be quite large to change our observed associations. Lastly, the chronic conditions group may be heterogenous and each component may differ in their association with CP and in the mediating effect of pre-term birth.

We provide prospective, population-based evidence that several maternal pre-existing chronic illness and pregnancy disorders are associated with an increased risk of CP in offspring. Pre-term delivery mediated a substantial part of the observed effect but our mediation analysis also suggests a direct effect acting independently of early birth or SGA status at birth. The absolute risk of CP and the PAF associated with pre-pregnancy and pregnancy disorders are small but understanding the pathophysiological pathways underlying their effects may help in devising preventive strategies.

## Ethics approval

Our study was approved by the Ethics Review Authority in Sweden (No. 2022–01155-02).

## Supplementary Material

dyad106_Supplementary_Data

## Data Availability

The data underlying this article cannot be shared publicly due to privacy and ethical restrictions. The data will be shared on reasonable request to the corresponding author.
