# Supplementary material for: Pre-pregnancy and pregnancy disorders, pre-term birth and the risk of cerebral palsy: a population-based study
Source: Int J Epidemiol. 2023 Jul 26;52(6):1766–73. doi: 10.1093/ije/dyad106 (PMC10749773; doi:10.1093/ije/dyad106)
Supplement: dyad106_Supplementary_Data [file dyad106_supplementary_data.docx]

**Supplementary analyses**

| **Table S1. ICD-9 and ICD-10 codes for maternal conditions.** | | |
| --- | --- | --- |
| **Maternal conditions** | **ICD 10** | **ICD 9** |
| **Chronic disorders** |  |  |
| Inflammatory bowel disease | K50, K51 | 555, 556 |
| Celiac disease | K90.0 | 579 |
| Rheumatoid arthritis | M05-M09 | 714 |
| Lupus erythematosus | M32 | 710 |
| Multiple sclerosis | G35 | 340 |
| Thyroid disorder | E00–E07 | 240-246 |
| Epilepsy | G40-G41 | 345 |
| Migraine | G43 | 346 |
| Asthma, Bronchiectasis | J45-J46, J47 | 493, 494 |
| Chronic kidney disease | N17-N19 | 584-586 |
| **Cardiovascular and metabolic disorders** |  |  |
| Ischemic heart disease | I20-I25 | 410-414 |
| Chronic heart disease | I05-I09, I34-I37, I42, I43, I50 | 394-397, 424, 425, 428 |
| Cerebrovascular diseases | I60-I69 | 430-438 |
| Chronic hypertension* | O10, O11, I10-15 | 401-5, 642C, 642H |
| Type 1 and 2 Diabetes | E10, O24.0, E11, O24.1 | 250, 648.0 |
| **Mental disorders** |  |  |
| Anxiety and stress-related disorders | F40, F41, F43 | 300.0, 300.2, 308, 309 |
| Depression and other mood disorders | F32, F33, F34 (excluding F34.0), F38, F39 | 296.1, 298.0, 300.4, 311 |
| Eating disorders | F50.0-F50.3, F50.9 | 307.1, 307.5 |
| Bipolar disorders | F25.0, F30, F31, F34.0 | 296.0, 296.2, 296.3, 296.4, 296.5, 296.6 |
| Schizophrenia and other psychotic disorders | F20, F21, F22, F23, F24, F25 (excluding F25.0), F28, F29 | 295 (minus 295.5), 297, 298 (minus 298.0) |
| **Pre-pregnancy Obesity** | body mass index [BMI] ≥30 kg/m^2^ |  |
| **Pregnancy complications** |  |  |
| Antepartum hemorrhage |  |  |
| *Placenta previa* | O44 | 661 |
| *Placental abruption* | O45 | 659.1 |
| *Antepartum hemorrhage* | O46 | 641.3, 641.2, 641.8, 641.9 |
| Premature rupture of membranes | O42.1 | 658.1 |
| Preeclampsia and eclampsia | O14 and O15 | 642.4, 642.5, 642.6 |
| Gestational diabetes | O24.4 | 648.8 |
| Major malformation | ICD-10: Q00-Q99, excluded minor malformation ICD-10: Q17.0, Q17.5, Q18.0, Q18.1, Q25.0, Q27.0, Q31.5, Q32.0, Q52.3, Q53.0 to Q53.9, Q66.5 to Q66.9, Q69.0 to Q69.9, Q70.0 to Q70.9, Q76.0, Q79.9, Q82.5, and Q82.9 |  |

| **Table S2. Maternal Chronic disorders by type** | | |
| --- | --- | --- |
| **Disorders** | **No.** | **%** |
| **Depression** | 76872 | 3.74 |
| **Asthma** | 60821 | 2.96 |
| **Thyroid disorder** | 41452 | 2.02 |
| **Migraine** | 32799 | 1.60 |
| **Chronic hypertension** | 23858 | 1.16 |
| **Diabetes** | 22673 | 1.10 |
| **Anxiety** | 21788 | 1.06 |
| **Eating disorders** | 21788 | 1.06 |
| **Inflammatory bowel disease** | 14775 | 0.72 |
| **Celiac disease** | 10863 | 0.53 |
| **Bipolar disorder** | 9908 | 0.48 |
| **Epilepsy** | 9836 | 0.48 |
| **Rheumatoid arthritis** | 8199 | 0.40 |
| **Psychosis** | 5142 | 0.25 |
| **Multiple Sclerosis** | 2946 | 0.14 |
| **Chronic heart disease** | 2827 | 0.14 |
| **Cerebrovascular disease** | 2606 | 0.13 |
| **SLE** | 1848 | 0.09 |
| **Chronic kidney disease** | 1637 | 0.08 |
| **Ischemic heart disease** | 444 | 0.02 |

| **Table S3. Pre-existing maternal chronic disorders and pregnancy-related complications and risk of preterm birth (<37 weeks) and small for gestational age. Singleton offspring in Sweden singleton births without congenital malformation 1999-2019** | |
| --- | --- |
| **Maternal Disorders** | **Relative risk (95 % CI)*** |
| **Preterm birth** |  |
| Chronic cardiovascular & metabolic disorders^a^ | 2.95 (2.86-3.04) |
| Mental disorders^b^ | 1.32 (1.29-1.35) |
| Other chronic disorders^c^ | 1.33 (1.30-1.36) |
| Obesity (BMI ≥30 kg/m^2^) | 1.48 (1.39-1.57) |
| Gestational diabetes | 1.54 (1.47-1.61) |
| Preeclampsia | 4.47 (4.38-4.56) |
| Antepartal hemorrhage^d^ | 8.54 (8.37-8.72) |
| **Small for gestational age** |  |
| Chronic cardiovascular & metabolic disorders^a^ | 1.37 (1.31-1.42) |
| Mental disorders^b^ | 0.99 (0.96-1.01) |
| Other chronic disorders^c^ | 1.09 (1.07-1.11) |
| Obesity (BMI ≥30 kg/m^2^) | 0.82 (0.77-0.87) |
| Gestational diabetes | 0.71 (0.67-0.75) |
| Preeclampsia | 3.21 (3.15-3.27) |
| Antepartal hemorrhage^d^ | 1.56 (1.50-1.63) |
| ^a^Chronic cardiovascular & metabolic diseases: any of Type 1 and 2 Diabetes, ischemic heart disease, chronic heart disease, cerebrovascular diseases, and chronic hypertension. | |
| ^b^Mental disorder includes any of anxiety and stress-related disorders, Depression and other mood disorders, Eating disorders, Bipolar disorders, Schizophrenia and other psychotic disorders | |
| ^c^Other chronic disorders include: asthma, thyroid disorder, inflammatory bowel disease, epilepsy, multiple sclerosis, migraine, celiac disease, rheumatoid arthritis, lupus erythematosus, and chronic kidney disease | |
| ^d^Antepartal hemorrhage include: placenta previa, placental abruption, and other reasons for antepartum bleeding | |
| * Adjusted for maternal age at childbirth, parity, educational level, country of birth, smoking during pregnancy, cohabitation with a partner, year of delivery and child's sex | |

| **Table S4. Robustness to unmeasured confounding (E-values) of the total effect and natural indirect effect adjusted risk ratios for the relation between maternal disorders and cerebral palsy. Liveborn singleton infants in Sweden, 1999–2019** | | | | | | |
| --- | --- | --- | --- | --- | --- | --- |
| **Preterm birth mediator** | **Risk Ratio (95% CI) for outcomes** | | | | | |
|  | **Natural indirect effect** | **E-value for RR** | **E-value for lower 95% CI** | **Total effect** | **E-value for RR** | **E-value for lower 95% CI** |
| **Cerebral Palsy** | | | | | | |
| Chronic cardiovascular & metabolic disorders | 1.29 (1.17-1.41) | 1.90 | 1.62 | 1.88 (1.52-2.25) | 3.17 | 2.41 |
| Other chronic disorders | 1.07 (1.06-1.09) | 1.34 | 1.31 | 1.24 (1.08-1.39) | 1.79 | 1.37 |
| Mental disorders | 1.07 (1.05-1.09) | 1.34 | 1.28 | 1.25 (1.09-1.41) | 1.81 | 1.40 |
| Obesity (BMI ≥30 kg/m^2^) | 1.04 (1.03-1.05) | 1.24 | 1.21 | 1.36 (1.22-1.49) | 2.06 | 1.74 |
| Preeclampsia | 1.41 (1.27-1.55) | 3.68 | 3.04 | 2.13 (1.82-2.45) | 3.68 | 3.04 |
| Antepartal hemorrhage | 1.96 (1.62-2.30) | 3.33 | 2.62 | 5.77 (4.93-6.61) | 11.02 | 9.33 |

| **Table S5. Pre-existing maternal chronic disorders and pregnancy-related complications and hazard ration (95% CI) for cerebral palsy. Live-born singleton offspring in Sweden, singleton births without congenital malformation, 1999-2019** | | | | | | |
| --- | --- | --- | --- | --- | --- | --- |
| **Maternal disorders** | **No· of children** |  | **No. of cases** | **Rate per 1000 person-time** | **Hazard ratio (95 % CI)** | |
|  |  | **Person time** |  |  | **Model 1^e^** | **Model 2^f^** |
| Chronic cardiovascular & metabolic disorders^a^ | 38470 | 371493 | 128 | 0.34 | 2.08 (1.74-2.48) | 1.90 (1.56-2.30) |
| Other chronic disorders^c^ | 177105 | 1413362 | 320 | 0.23 | 1.19 (1.06-1.33) | 1.25 (1.11-1.41) |
| Mental disorders^b^ | 159341 | 1204960 | 305 | 0.25 | 1.28 (1.14-1.44) | 1.27 (1.11-1.44) |
| Obesity (BMI ≥30 kg/m^2^) | 234588 | 2294751 | 499 | 0.22 | 1.35 (1.23-1.48) | 1.36 (1.23-1.50) |
| Gestational diabetes | 26695 | 231179 | 55 | 0.24 | 1.37 (1.05-1.78) | 1.26 (0.95-1.67) |
| Preeclampsia | 55670 | 602895 | 210 | 0.35 | 2.31 (2.01-2.65) | 2.15 (1.85-2.50) |
| Antepartum hemorrhage^d^ | 22375 | 233712 | 221 | 0.95 | 6.32 (5.52-7.24) | 5.92 (5.12-6.85) |
| ^a^Chronic cardiovascular & metabolic diseases: any of Type 1 and 2 Diabetes, ischemic heart disease, chronic heart disease, cerebrovascular diseases, and chronic hypertension. | | | | | | |
| ^b^Mental disorder includes any of anxiety and stress-related disorders, Depression and other mood disorders, Eating disorders, Bipolar disorders, Schizophrenia and other psychotic disorders | | | | | | |
| ^c^Other chronic disorders include: asthma, thyroid disorder, inflammatory bowel disease, epilepsy, multiple sclerosis, migraine, celiac disease, rheumatoid arthritis, lupus erythematosus, and chronic kidney disease | | | | | | |
| ^d^Antepartal hemorrhage include: placenta previa, placental abruption, and other reasons for antepartum bleeding | | | | | | |
| ^e^Unadjusted model. |  |  |  |  |  |  |
| ^f^Model 2 adjusted for maternal age at childbirth, parity, educational level, country of birth, smoking during pregnancy, cohabitation with a partner, year of delivery, and child's sex | | | | | | |

| **Table S6. Multiple-imputation analysis of the associations between pre-existing maternal chronic disorders and pregnancy-related complications and risk of cerebral palsy. Singleton offspring in Sweden, singleton births without congenital malformation, 1999-2019** | |
| --- | --- |
| **Maternal Disorders** | **Relative risk (95 % CI)*** |
| Chronic cardiovascular & metabolic disorders^a^ | 2.07 (1.74-2.47) |
| Mental disorders^b^ | 1.31 (1.16-1.48) |
| Other chronic disorders^c^ | 1.28 (1.14-1.44) |
| Obesity (BMI ≥30 kg/m^2^) | 1.32 (1.20-1.45) |
| Gestational diabetes | 1.24 (0.95-1.62) |
| Preeclampsia | 2.16 (1.88-2.49) |
| Antepartal hemorrhage^d^ | 5.87 (5.12-6.72) |
| ^a^Chronic cardiovascular & metabolic diseases: any of Type 1 and 2 Diabetes, ischemic heart disease, chronic heart disease, cerebrovascular diseases, and chronic hypertension. | |
| ^b^Mental disorder includes any of anxiety and stress-related disorders, Depression and other mood disorders, Eating disorders, Bipolar disorders, Schizophrenia and other psychotic disorders | |
| ^c^Other chronic disorders include: asthma, thyroid disorder, inflammatory bowel disease, epilepsy, multiple sclerosis, migraine, celiac disease, rheumatoid arthritis, lupus erythematosus, and chronic kidney disease | |
| ^d^Antepartal hemorrhage include: placenta previa, placental abruption, and other reasons for antepartum bleeding | |
| * Adjusted for maternal age at childbirth, parity, educational level, country of birth, smoking during pregnancy, cohabitation with a partner, year of delivery and child's sex | |

| **Table S7. Pre-existing maternal chronic disorders and pregnancy-related complications diagnosed within 5 years prior to pregnancy and risk ratio (95% CI) for cerebral palsy. Live-born singleton offspring in Sweden, singleton births without congenital malformation, 1999-2019** | | | |
| --- | --- | --- | --- |
| **Maternal disorders** |  | **Risk ratio (95 % CI)** | |
|  | **No. of CP (%)** | **Model 1^d^** | **Model 2^f^** |
| Chronic cardiovascular & metabolic disorders^a^ | 114 (0.33) | 1.99 (1.65-2.40) | 1.85 (1.51-2.28) |
| Other chronic disorders^c^ | 223 (0.19) | 1.14 (1.00-1.31) | 1.28 (1.11-1.48) |
| Mental disorders^b^ | 212 (0.20) | 1.18 (1.03-1.36) | 1.21 (1.04-1.41) |
| ^a^Chronic cardiovascular & metabolic diseases: any of Type 1 and 2 Diabetes, ischemic heart disease, chronic heart disease, cerebrovascular diseases, and chronic hypertension. | | | |
| ^b^Mental disorder includes any of anxiety and stress-related disorders, Depression and other mood disorders, Eating disorders, Bipolar disorders, Schizophrenia and other psychotic disorders | | | |
| ^c^Other chronic disorders include: asthma, thyroid disorder, inflammatory bowel disease, epilepsy, multiple sclerosis, migraine, celiac disease, rheumatoid arthritis, lupus erythematosus, and chronic kidney disease | | | |
| ^d^Unadjusted model. |  |  |  |
| ^f^Model 2 adjusted for maternal age at childbirth, parity, educational level, country of birth, smoking during pregnancy, cohabitation with a partner, year of delivery, and child's sex | | | |
